# The association of thigh myosteatosis with lower cognitive function in older cancer survivors

**DOI:** 10.1007/s11357-025-01857-6

**Published:** 2025-09-05

**Authors:** Brendan L. McNeish, Brian Kunzer, Sarah G. Bell, Caterina Rosano

**Affiliations:** 1 Department of Physical Medicine and Rehabilitation, University of Michigan, Ann Arbor, MI USA; 2 Department of Physical Medicine and Rehabilitation, University of Pittsburgh, Pittsburgh, PA USA; 3 Department of Epidemiology, School of Public Health, University of Pittsburgh, Pittsburgh, PA USA; 4 Department of Obstetrics and Gynecology, University of Michigan, Ann Arbor, MI USA

**Keywords:** Cognitive function, Myosteatosis, Cancer survivor, Rehabilitation, Muscle-brain crosstalk

## Abstract

**Introduction:**

Cancer is associated with accelerated aging, including changes in muscle composition and cognition. However, the relationship between myosteatosis and cognitive function has not been investigated in older cancer survivors. This study evaluated the association between myosteatosis and cognitive function in this population.

**Methods:**

The sample included 75 cancer survivors (age 76–85; 65% men; 31% Black; 41% prostate cancer; 21% breast cancer) from the Health ABC study who developed cancer within the first five years, completed CT imaging at Year 6, and cognitive testing at Year 10. Thigh intermuscular fat area (myosteatosis) was measured by CT at Year 6. Cognitive function was assessed using the Digit Symbol Substitution Test (DSST) and Modified Mini-Mental Status Exam (3MS) at Years 5 and 10. Multivariable models adjusted for demographics, education, cognitive function, and thigh muscle area at Year 5. Sensitivity analyses adjusted for leg strength, race, dementia risk factors, BMI, abdominal visceral fat, and thigh subcutaneous fat. LASSO regression identified key predictors of DSST and 3MS scores.

**Results:**

Greater thigh myosteatosis at Year 6 was associated with lower DSST (B =  − 0.212, *p* < 0.05) and 3MS (B =  − 0.145, *p* < 0.05) scores at Year 10. Associations remained significant after adjustment for strength, dementia risk, and adiposity. LASSO identified race, education, Year 5 cognition, and myosteatosis as key predictors for DSST as well as thigh muscle area and physical activity for 3MS.

**Discussion:**

Thigh myosteatosis is independently associated with lower cognitive performance in older cancer survivors and may represent a rehabilitation target to improve cognitive outcomes.

**Graphical Abstract:**

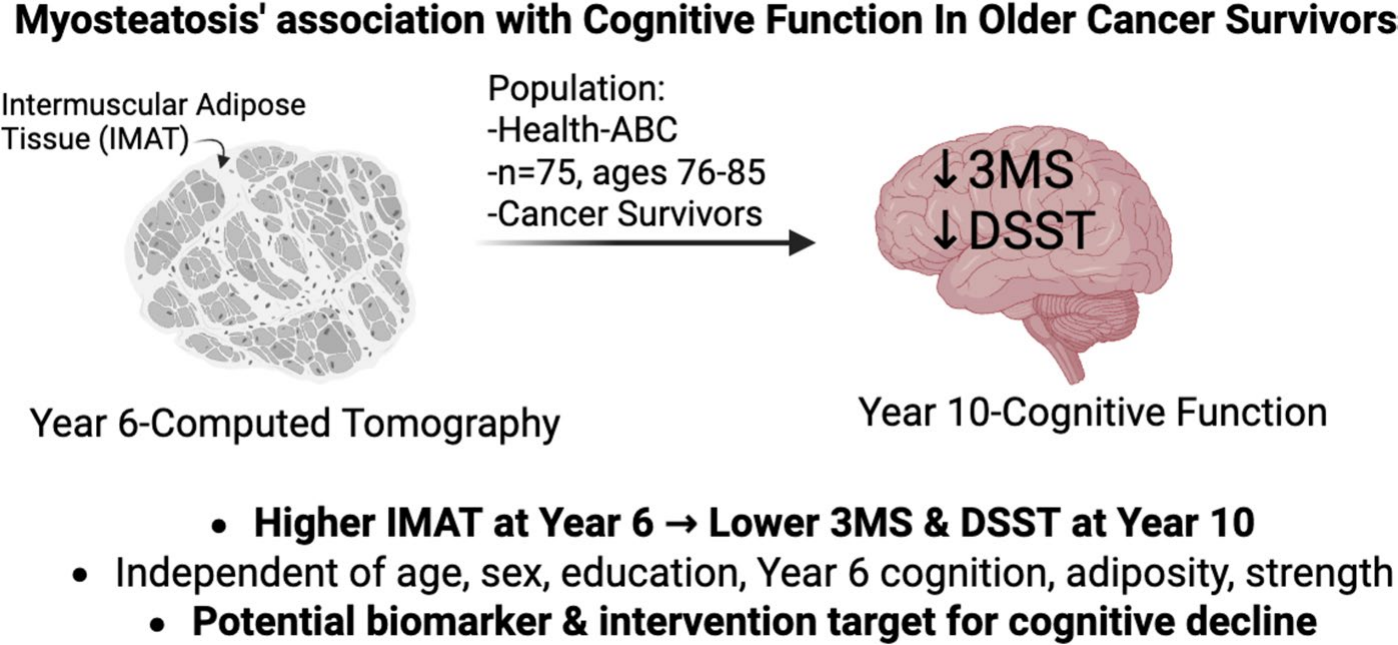

**Supplementary Information:**

The online version contains supplementary material available at 10.1007/s11357-025-01857-6.

## Introduction

Cancer-related cognitive impairment is a common adverse effect of cancer and its treatment in older cancer survivors [1]. In particular, older cancer survivors may be at increased risk for developing dementia [2]. Cognitive function in older cancer survivors is linked to independence, quality of life, mobility, and falls [3–7]. Therefore, identifying the drivers of cognitive function may enable early detection of at-risk individuals and inform targeted interventions to improve cognitive health, reduce dementia risk, and improve overall cancer survivorship.

Myosteatosis is emerging as a novel risk factor for cognitive impairment among older adults [8–10]. Myosteatosis refers to the accumulation of fat within and around skeletal muscle, encompassing (a) intermuscular adipose tissue (IMAT)—extracellular fat deposits between a muscle and its overlying fascia or neighboring muscles, (b) intramuscular adipose tissue—fat between muscle fibers within a single muscle, and (c)intramyocellular lipids—lipid droplets within myocytes [11]. Although these fat depots are distinct and represent different aspects of myosteatosis, they are correlated and measures of myosteatosis. IMAT and intramuscular adipose tissue are the myosteatosis depots most commonly studied, as they can be measured with imaging methods such as computed tomography and magnetic resonance imaging [12]. Both IMAT and intramuscular adipose tissue, as components of myosteatosis, have been linked to cognitive function and brain health [8–10, 13].

Older cancer survivors are especially vulnerable to changes in both muscle function and morphology, with clinical syndromes ranging from cachexia to sarcopenia [14]. In older cancer survivors, muscle loss and myosteatosis have been associated with survival, treatment-related toxicity, and declines in physical function [15, 16]. However, the relationship between myosteatosis and cognitive function in older cancer survivors remains largely unexplored. If myosteatosis is associated with future cognitive function in this population, then interventions targeting myosteatosis may offer a pathway to improve cognitive outcomes.

The objective of this study was to investigate the association between skeletal myosteatosis and cognitive function in older cancer survivors. We used data from the Health ABC cohort, which enrolled older adults and tracked cancer incidence throughout the study. Myosteatosis was assessed via computed tomography (CT) to measure IMAT at Year 6, a method recognized as the gold standard for measuring myosteatosis. Cognitive outcomes, including processing speed via the Digit Symbol Substitution Test (DSST) and general cognitive function via the Modified Mini-Mental State Exam (3MS), were measured at Year 10. Covariates included established dementia risk factors and measures of general and central adiposity. We hypothesized that higher levels of thigh myosteatosis at Year 6 would be associated with worse cognitive performance at Year 10, based on similar findings in cancer-free older adult populations.

## Methods

### Population and general study design

The Health, Aging and Body Composition (Health ABC) Study enrolled 3,075 adults aged 70–79 from Memphis, Tennessee, and Pittsburgh, Pennsylvania, to investigate changes in body composition, functional limitation, disability, and mortality. Participants were eligible if they had no difficulty walking ¼ mile or climbing 10 stairs. At baseline (1997–1998), cancer history was recorded, and individuals undergoing active cancer treatment were excluded. Incident cancer diagnoses were tracked annually and affected participants remained in the study. For this secondary analysis, only cancer survivors were included (a subset of Health ABC). Cancer survivors were defined as participants with a new cancer diagnosis (excluding skin cancer) within the first five years of enrolling in the study. Cognitive assessments (DSST and 3MS) were conducted at Years 5 and 10. Body composition (thigh intermuscular adipose tissue [IMAT], visceral and subcutaneous fat, and muscle area) was assessed by CT at Year 6. The primary independent variable was thigh IMAT (Year 6); the main dependent variables were DSST and 3MS scores (Year 10).

Participants included had Year 6 CT imaging and Year 10 cognitive testing. Of 3,075 participants, 373 were incident cancer survivors; after excluding those without CT imaging at Year 6 (*n* = 255) or without cognitive testing at Years 5 and 10, the final analytic sample included 75 participants. Missing data were primarily due to death or disability. All participants provided written informed consent. Protocols were approved by the institutional review boards of the University of Tennessee Memphis, the University of Pittsburgh, and the University of California San Francisco.

### Independent variable

Thigh myosteatosis was measured as the intermuscular fat area of the thigh muscles, including the quadriceps and hamstrings. Thigh CT scans were obtained at Year 6 at the Pittsburgh site using a 9800 Advantage Scanner (General Electric, Milwaukee, WI) and at the Memphis site using either a Somatom Plus 4 (Siemens, Erlangen, Germany) or a Picker PQ 2000S (Marconi Medical Systems, Cleveland, OH), with high reliability [17]. Thigh IMAT was separated from subcutaneous fat by tracing the deep fascial plane surrounding the thigh muscles, using muscle attenuation values as previously described [8]. The total area of nonfat, nonbone tissue within the fascial border quantified thigh muscle area (cm^2^).

### Dependent variables

The DSST is a paper-and-pencil test of executive function and processing speed, where participants match symbols to digits using a key, completing as many correct matches as possible in 90 s [18]. Scores reflect the number of correct responses with higher scores indicating better performance. The DSST was administered at Years 5 and 10.

The 3MS is a validated cognitive screening tool (0–100 scale) assessing orientation, attention, memory, construction, language, verbal fluency, and conceptualization [19]. Higher scores indicate better global cognitive function. The 3MS was administered at Years 5 and 10.

### Other adiposity measures

Body weight and height were measured and used to calculate body mass index (BMI) as a measure of general adiposity. Abdominal visceral and subcutaneous fat areas were measured by CT at the L4–L5 level in Year 6, with fat area calculated by multiplying tissue pixel counts by pixel area using ILD development software (RSI Systems, Boulder, CO). Visceral fat was manually separated from subcutaneous fat by tracing the fascial plane of the internal abdominal wall, a measure of central adiposity. Thigh subcutaneous fat area was measured from cross-sectional CT scans using anatomical landmarks and radiographic density.

### Covariates for cognitive decline

At Year 1, self-reported age, sex, race/ethnicity, and years of education (categorized as postsecondary or less) were collected. Participants identified race/ethnicity from a list including Asian/Pacific Islander, Black/African American, White/Caucasian, Latino/Hispanic, “do not know,” “other” (specified), or “refused.” Apolipoprotein E4 (APOE4) status was determined via standard SNP genotyping. Diabetes and hypertension were assessed by self-report, medication use, or Health Care Finance Administration diagnoses. Total weekly energy expenditure (kcal/kg/week) was estimated from self-reported physical activity over the prior 12 months.

Right knee extension strength was measured using an isokinetic dynamometer (Kin-Com 125 AP; Chattanooga, TN) at 60°/s, with joint angles set from 90° to 30° after the average of three acceptable and reproducible trials out of a maximum of six attempts [20].

### Statistical analysis

Descriptive statistics were calculated to summarize demographic characteristics, dementia risk factors, muscle strength, and cognitive performance for the analytic sample (*n* = 75). Results are presented as percentages, means ± standard deviations, or medians with interquartile ranges, depending on variable type and distribution. Histogram review showed DSST scores were normally distributed, while 3MS scores were negatively skewed. Accordingly, both Pearson and Spearman correlation coefficients were used to examine associations between myosteatosis, other fat depots, and cognitive outcomes.

Primary multivariable linear regression models were constructed using thigh intermuscular fat area as the independent variable and DSST and 3MS scores at Year 10 as dependent variables. The base model included covariates selected a priori: age, gender, education, cognitive function at Year 5, and thigh muscle area. Sensitivity analyses were performed by sequentially adjusting the base model for additional covariates, including knee extension strength, race, dementia risk factors (diabetes, hypertension, APOe4 status, and physical activity), and additional fat depots (BMI, visceral fat area, and thigh subcutaneous fat area).

In parallel, to address the modest sample size and relatively large number of candidate predictors, we applied least absolute shrinkage and selection operator (LASSO) regression for variable selection and regularization. LASSO was implemented using tenfold cross-validation across 100 lambda values to identify the most predictive variables. Variables with nonzero coefficients selected by LASSO were included in a separate standard multivariable linear regression model to estimate interpretable coefficients and 95% confidence intervals. All analyses were performed using Stata version 19.5, with *p* < 0.05 considered statistically significant.

## Results

The characteristics of the cancer survivor cohort are presented in Table 1. Overall, the participants had a mean age of 79 ± 3 years at Year 6, with 65% male and 69% identifying as White. Additionally, 67% of participants had completed postsecondary education, while 32% had diabetes and 43% had hypertension. Regarding cancer history, survivors most commonly reported prostate cancer (41%), followed by other cancers (26%), breast cancer (21%), and colon cancer (12%).

**Table 1:** Characteristics of 75 Older Cancer Survivors

	Mean
Age	79.4 (2.9)
Male	65% (49)
White	69% (52)
Education (postsecondary)	67% (50)
APOE4 allele	23% (16)
Cancer Type	
Prostate	41% (31)
Breast	21% (16)
Colon	12% (9)
Other	26% (19)
BMI	26.2 (3.9)
Diabetes	32% (24)
Hypertension	43% (32)
Physical Activity (kcal/kg/week)	77.1 (50.8)
Knee Extension Force (Newtons)	311 (108)
3MS 5	92.6 (6.61)
3MS 10	91.2 (7.43)
DSST 5	37.5 (12.2)
DSST 10	35.2 (11.4)

BMI = Body mass index,3MS5 = Modified Mini-Mental State Examination score at Year 5, 3MS10 = Modified Mini-Mental State Examination score at Year 10, DSST5 = Digit Symbol Substitution Test score at Year 5, DSST10 = Digit Symbol Substitution Test score at Year 10

Table 2 presents the means and standard deviations of myosteatosis and other adiposity measures, which were collected via computed tomography, along with their correlations with DSST and 3MS scores. Thigh myosteatosis demonstrated significant negative correlations with both DSST and 3MS scores. In contrast, thigh muscle area and none of the other adiposity measures were significantly correlated with DSST or 3MS.

**Table 2:** Mean Values of muscle and adiposity measures at Year 6 and their correlation with Digit Symbol Substitution test and Mini Mental Status Score Year 10

	Mean (Standard Deviation)	Pearson Correlation with DSST	Spearman Correlation with 3MS
Thigh Intermuscular Fat Area (cm2)	24.3 (10.8)	−0.34**	−0.35**
Thigh Muscle Area (cm2)	219.4 (51.4)	−0.06	.07
Thigh Subcutaneous Fat Area (cm2)	133.0 (62.2)	−0.01	−0.09
Abdominal Visceral Fat Area (cm2)	144.1 (76.0)	−0.07	−0.09
BMI	26.8 (4.1)	−0.16	−0.15

*p < 0.05

**p < 0.01

Cm2 = the square of centimeters

BMI = Body mass index

Thigh myosteatosis was significantly negatively associated with DSST (B = −0.214, *p* = 0.04) and 3MS (B = −0.145, *p* = 0.01) after adjusting for age, gender, education, cognitive function at Year 5, and thigh muscle area in base multivariable models (Table 3). Adding knee extension strength did not increase explained variance for either cognitive outcome and was not independently associated with DSST or 3MS. Thigh muscle area was not associated with DSST but was positively associated with 3MS (B = 0.039, *p* = 0.03), remaining significant after adjustment for knee strength (B = 0.051, *p* = 0.01).

**Table 3:** Multivariable models of the association between thigh myosteatosis and cognitive function (Digit Symbol Substitution Test and Modified Mini-Mental State scores), with adjustment for muscle strength. Unstandardized beta coefficients (B), 95% confidence intervals (CI), and p-values are shown

Variable	DSST	MMS	DSST-Adjusted	MMS-Adjusted
Thigh Intermuscular Fat Area (cm2)	−0.214 (−0.416,−0.012) *p* = 0.04	−0.145 (−0.253,−0.037) *p* = 0.01	−0.228 (−0.443,−0.013) *p* = 0.04	−0.161 (−0.274,−0.048) *p* = 0.01
Thigh Muscle Area (cm2)	0.032 (−0.040,0.010) *p* = 0.43	0.039 (0.004,0.074) *p* = 0.03	0.009 (−0.067,0.086) *p* = 0.81	0.052 (0.011,0.093) *p* = 0.01
Age	−0.247 (−0.971,0.447) *p* = 0.50	0.296 (−0.109,0.700) *p* = 0.15	−0.258 (−1.01,0.485) *p* = 0.49	0.225 (−0.186,0.636) *p* = 0.28
Gender	1.01 (−5.36,7.39) *p* = 0.75	2.34 (−1.05,5.73) *p* = 0.17	1.46 (−5.17,8.08) *p* = 0.66	1.71 (−1.75,5.17) *p* = 0.33
Postsecondary Education	2.48 (−2.13,7.08) *p* = 0.29	0.318 (−2.34,2.97) *p* = 0.81	1.89 (−2.88,6.67) *p* = 0.43	0.350 (−2.35,3.04) *p* = 0.80
Cognitive Function at Year 5†	0.594 (0.420,0.768) *p* < 0.001	0.912 (0.734,1.09) *p* < 0.001	0.582 (0.404,0.760) *p* < 0.001	0.934 (0.753,1.12) *p* < 0.001
KE Strength			0.014 (−0.014,0.040) *p* = 0.31	−0.012 (−0.028,0.004) *p* = 0.16
Adjusted R2	0.53	0.75	0.53	0.75
R2	0.57	0.77	0.58	0.78

†For models for DSST, cognitive function at Year 5 was DSST at Year 5 and for models for 3MS, cognitive function was 3MS at Year 5

KE = knee extensor; DSST = Digit Symbol Substitution Test; 3MS = Modified Mini-Mental State Exam; Cm2 = the square of centimeters

Sensitivity analyses adjusting for race, dementia risk factors (hypertension, diabetes, APOE4, physical activity), and adiposity measures (BMI, thigh subcutaneous fat area, and visceral fat area) are shown in Supplementary Tables 1 and 2. The association between thigh myosteatosis and DSST remained significant after adjusting for race and dementia risk factors, but was attenuated to borderline significance with the addition of APOE4 (B = −0.209, *p* = 0.056). The association between thigh myosteatosis and DSST also remained significant after adjusting for BMI and visceral fat area, but was reduced to borderline significance with the addition of subcutaneous fat area (B = −0.219, *p* = 0.056). Thigh Muscle Area continued to not be significant with its association with DSST after adjusting for any of dementia risk factors and other measures of adiposity.

The association between thigh myosteatosis and 3MS remained robust across all sensitivity models, with minimal attenuation (< 10%). None of the additional dementia or adiposity covariates were independently significant (all p > 0.05), though physical activity approached significance for 3MS (B = 0.020, *p* = 0.06). Thigh muscle area remained positively associated with 3MS except for adjustments for BMI (B = 0.38, *p* = 0.08)and physical activity (B = 0.34, *p* = 0.06) when adjusting for dementia risk factors and measures of adiposity.

LASSO-optimized models are presented in Table 4. For DSST, the model selected thigh myosteatosis, race, education, and DSST at Year 5 as predictors (lambda = 1.65), resulting in four retained variables. For 3MS, the model retained thigh myosteatosis, thigh muscle area, race, education, 3MS at Year 5, and total physical activity (lambda = 0.544), resulting in six retained variables. In the final DSST model, thigh myosteatosis remained negatively associated (B = −0.180, 95% CI: −0.357, −0.003, *p* = 0.04), while DSST at Year 5 was positively associated (B = 0.565, 95% CI: 0.395, 0.735, *p* < 0.001); race and education were not significant. In the final 3MS model, thigh myosteatosis remained negatively associated (B = −0.115, 95% CI: −0.213, −0.017, *p* = 0.02); 3MS at Year 5 was positively associated (B = 0.906, 95% CI: 0.715, 1.10, *p* < 0.001); and physical activity was also positively associated (B = 0.022, 95% CI: 0.002, 0.042, *p* < 0.03). Race, education, and thigh muscle area were not significantly associated with 3MS at Year 10.

**Table 4:** Multivariable linear regression models for DSST and 3MS outcomes in older cancer survivors: Variables selected via LASSO regression with unstandardized beta coefficients, 95% confidence intervals, and p-values

Variable	DSST	3MS
Thigh Intermuscular Fat Area (cm2)	−0.180 (−0.357,−0.003) *p* = 0.04	−0.115 (−0.213,−0.017) *p* = 0.02
Black Race	−2.13 (−10.4,0.089) *p* = 0.32	−1.15 (−3.70,−1.41) *p* = 0.37
Education postsecondary	2.67 (−1.59,6.93) *p* = 0.21	−0.300 (−3.00,2.40) *p* = 0.83
Cognitive Function at Y5	0.565 (0.395,0.735) *p* < 0.001	0.906 (0.715,1.10) *p* < 0.001
Thigh Muscle Area		0.021 (−0.002,0.044) *p* = 0.422
Physical Activity		0.022 (0.002, 0.042) *p* = 0.03
R2	0.57	0.78
Adjusted R2	0.54	0.75

## Discussion

In this sample of older cancer survivors, greater thigh myosteatosis was associated with lower processing speed and overall cognitive function, as measured by the DSST and 3MS, respectively. While thigh muscle area was associated with 3MS in the base multivariable model, it was not associated with DSST in any of the models, and association with 3MS was not significant after adjustment for BMI or physical activity. Additionally, cognitive function at year 5 was significantly associated with lower cognitive scores at year 10 for both DSST and 3MS, while total physical activity was associated with 3MS in the final multivariable models. Taken together, these findings suggest that thigh myosteatosis may provide unique clinical insight as both a diagnostic marker and a potential rehabilitation target for cognitive decline in older cancer survivors.

Our finding that greater skeletal myosteatosis is associated with lower cognitive function in older cancer survivors is supported by the geriatric literature which is an important reference given that cancer survivors undergo accentuated and accelerated aging [21, 22]. In cancer-free older adults, increased skeletal myosteatosis has been associated with poorer performance on both the 3MS, a measure of global cognitive function, and the DSST, a measure of processing speed [8, 9]. This relationship has been observed across diverse ethnic groups and in both men and women. In older cancer survivors, both muscle mass and myosteatosis have been linked to mortality and treatment-related toxicity [16, 23–25]. Notably, only myosteatosis—not muscle mass—has been associated with quality of life and physical function, including both objective assessments (e.g., Timed Up and Go) and patient-reported outcomes (e.g., instrumental activities of daily living) [15, 26]. Our findings extend this literature by demonstrating that, in older cancer survivors, thigh myosteatosis—but not thigh muscle mass—is associated with both DSST, a measure of processing speed, and 3MS, a measure of general cognitive function. Because functional outcomes such as walking and instrumental activities of daily living are influenced by both physical and cognitive capacities, our findings suggest that the previously observed relationship between myosteatosis and patient-reported function may be partially mediated by cognitive function [15]. Therefore, this study underscores the importance of measuring both physical and cognitive function when investigating the mechanisms through which myosteatosis influences mobility-related outcomes (e.g., walking), independence, and overall quality of life in older cancer survivors.

The association between thigh myosteatosis and cognitive function in our study remained significant even after adjusting for knee extension strength. Given the well-established relationship between muscle quality—particularly myosteatosis—and physical function, we included knee extension strength as it is a known key locomotor factor for mobility and walking [20, 27]. This finding aligns with existing literature in older, cancer-free adults, which has, in part, informed the hypothesis that the underlying aspects of muscle biology—including myosteatosis and the muscle secretome—may contribute to the strong relationship observed between physical and cognitive function. These insights support emerging evidence of muscle–brain crosstalk and the concept that skeletal muscle may play an active role in promoting or maintaining brain health [8, 28].

Our finding that higher levels of thigh myosteatosis remained associated with lower performance on both the 3MS and DSST, even after adjusting for dementia risk factors and other adiposity measures, is consistent with existing literature. In cancer-free older adults across multiple middle-aged and older adult cohorts, myosteatosis has been independently associated with measures of processing speed, executive function, and general cognitive performance [8–10, 29]. Similarly, in our older cancer survivor cohort, the association between thigh myosteatosis and both processing speed and general cognitive function was not attenuated and remained statistically significant after adjusting for established dementia risk factors—including diabetes mellitus, hypertension, APOE4 allele status, and total physical activity—as well as for other measures of adiposity, including BMI, thigh subcutaneous fat area, and abdominal visceral fat area. Collectively, these findings suggest that the observed link between myosteatosis and cognitive function in older cancer survivors may be driven by mechanisms beyond traditional dementia risk factors and may reflect effects specific to myosteatosis rather than general (e.g., BMI) or central (e.g., visceral fat) adiposity.

Thigh muscle area was not associated with DSST, but was associated with 3MS in our sample of older cancer survivors. However, older cancer-free peer populations have not consistently demonstrated an association between muscle area and 3MS, a validated measure of general cognitive function. It is possible that muscle size (i.e., muscle area) and muscle quality (i.e., myosteatosis) are both linked to cognitive function, but in different ways. In a large UK Biobank study of cancer-free older adults, muscle area was associated with performance on the Fluid Intelligence Test [13]. The Fluid Intelligence Test taps multiple domains of higher-order cognitive function, including memory, processing speed, flexibility, and reasoning, and may be more similar to the 3MS than the DSST. Given that myosteatosis reflects a combination of neurologic factors (e.g., loss of motor units via denervation) and cardiovascular factors (e.g., lipid accumulation in muscle depots), it may represent a marker of capacity for cognitive-motor control. Cognitive-motor control may be particularly important in tasks with time limits that test processing speed, visuomotor coordination, and sustained attention, such as the rapid symbol-number pairings required by the DSST. In contrast, muscle area is a more general characterization of muscle mass, reflecting body size and physical activity, but provides less information about contractile capacity or the internal makeup of muscle fibers. In our study, the association of thigh muscle area with 3MS was no longer significant (*p* = 0.06) after adjustment for physical activity. Additionally, cancer survivors experience accelerated aging, making older adult cohorts appropriate comparators; however, characteristics of muscle (e.g., muscle area and myosteatosis) may show both similarities and differences in their associations with brain health in cancer survivors compared to cancer-free populations. Our study adds to the literature on older cancer survivors by demonstrating an association of muscle area with 3MS but not with DSST. Future research is needed to clarify which specific aspects of cognitive function are most strongly linked to different features of muscle health.

Our study has several limitations. The relatively small sample of older cancer survivors limits statistical power and the ability to support complex multivariable models. To address this, we used sensitivity analyses and LASSO regression to facilitate multivariable modeling and improve interpretability of the association between thigh myosteatosis and cognitive function. Although Health ABC includes two assessments of myosteatosis (Years 1 and 6) and repeated cognitive testing (Years 1–10), we selected Year 6 for myosteatosis and Year 10 for cognitive outcomes to maximize the number of incident cancer cases with imaging data and to align with the administration of both the DSST and 3MS. Another limitation is the heterogeneity of cancer diagnoses and the lack of cancer treatment data in Health ABC. Specifically, the current study’s population consisted of 62% of prostate and breast whose treatment includes antihormonal therapy which could impact myosteatosis and/or cognitive function. A key limitation of our study is the absence of a non-cancer, healthy control group, which restricts our ability to determine whether changes in myosteatosis and cognition are specific to cancer or its treatments. Additionally, our small sample size and limited treatment information prevented stratification by cancer type or treatment. Future research should utilize larger, longitudinal cohorts with comprehensive treatment histories and include appropriate control groups, enabling more robust comparisons of myosteatosis and cognitive outcomes between cancer survivors and healthy individuals, as well as analyses by specific cancer types to address potential heterogeneity. Moreover, this study evaluated IMAT, a validated measure of myosteatosis, but future studies should also examine other intramuscular fat depots when investigating associations between myosteatosis and cognitive function. Additionally, because variables were selected using LASSO regression prior to model estimation as presented in Table 4, the resulting estimates and confidence intervals may be subject to post-selection inference bias. To address this, we first conducted standard multivariable analyses with sequential adjustment, followed by sensitivity analyses, and then applied LASSO regression to evaluate whether thigh myosteatosis was independently associated with cognitive function. Finally, survival bias may have influenced results, as 118 participants had CT imaging at Year 6 and cognitive testing at Year 5, but only 75 completed cognitive testing at Year 10; the remaining 43 were lost to follow-up due to death or disability.

Despite the noted limitations, the association between thigh myosteatosis and later cognitive function in older cancer survivors has meaningful clinical implications for both diagnostic assessment and rehabilitation. While it is well established that muscle mass, function, and quality—including myosteatosis—are relevant for cancer prognosis and physical function, our study suggests that myosteatosis may also serve as a potential biomarker for future cognitive decline [16]. Given that CT imaging is frequently used in cancer care, these scans could offer an opportunity to identify patients at elevated risk for cognitive impairment and refer them for early cognitive evaluation and intervention.

From a rehabilitation perspective, myosteatosis may represent a modifiable target not only for improving physical performance but also for enhancing cognitive outcomes. Emerging research has shown that strength training can improve cognitive function in both healthy older adults and individuals with Alzheimer’s disease [30]. Interest is growing in pharmacologic, nutritional, and structured exercise interventions—including resistance and aerobic training—to reduce myosteatosis and improve muscle health [28, 31]. These approaches may hold particular promise for older cancer survivors, a population uniquely vulnerable to a spectrum of muscle-related impairments ranging from cachexia to myosteatosis, and associated declines in both physical and cognitive function, including cancer-related cognitive impairment and increased dementia risk. To develop targeted interventions, future research should aim to identify the mechanistic drivers of myosteatosis that influence cognitive outcomes, including muscle–brain crosstalk. While part of this relationship may be mediated by improved physical function, there is growing evidence that the muscle secretome—particularly myokines and extracellular vesicles carrying bioactive molecules such as nucleic acids, lipids, and proteins—may play a direct role in influencing brain health [32]. Advancing our understanding of myosteatosis as a biomarker, rehabilitation target, and biologic mediator of muscle–brain communication may significantly enhance diagnostic precision and interventional strategies for older adults with cancer.

## Conclusion

Our findings suggest that higher levels of thigh myosteatosis are independently associated with lower cognitive function in older cancer survivors. Looking ahead, further research is needed to determine whether changes in myosteatosis predict future cognitive decline and to elucidate the biologic pathways by which myosteatosis mediates muscle–brain crosstalk. While insights can be drawn from studies in cancer-free older adults, the complex interaction between cancer, its treatments, and the aging process necessitates dedicated research in cancer survivor populations. These efforts will be critical to advancing our understanding of how muscle health contributes to cognitive aging and to developing targeted interventions that support both cognitive and physical function during cancer survivorship in older adults.

## Supplementary Information

Below is the link to the electronic supplementary material.

Supplementary file1 (DOCX 14 KB)

## Data Availability

Data is available upon reasonable request from Health ABC.

## Declarations

### Permission to reproduce material from other sources

NA.

### Clinical trial registration

NA.
