# Supplementary material for: The association of thigh myosteatosis with lower cognitive function in older cancer survivors
Source: GeroScience. 2025 Sep 5;48(3):3703–13. doi: 10.1007/s11357-025-01857-6 (PMC13356165; doi:10.1007/s11357-025-01857-6)
Supplement: Supplementary file 1 — Supplementary file1 (DOCX 14 KB) [file 11357_2025_1857_MOESM1_ESM.docx]

Supplementary Table 1: Sensitivity analyses evaluating the impact of dementia risk factors on the association between thigh myosteatosis and cognitive performance, as measured by the Digit Symbol Substitution Test (DSST) and Modified Mini-Mental Status Exam (3MS). The table presents the correlation coefficient, 95% confidence interval, and p-value for thigh myosteatosis and each added dementia risk factor in relation to cognitive outcomes.

| Variables | Models | | | | |
| --- | --- | --- | --- | --- | --- |
|  | DSST |  |  |  |  |
|  | Model1+Race | Model1+HTN | Model 1+DM | Model1+APOe4 | Model1+PA |
| Thigh Intermuscular Fat Area | -0.212  (-0.413,-0.011)  p=0.04 | -0.214  (-0.417,-0.011)  p=0.04 | -0.211  (-0.417,-0.006)  p=0.04 | -0.209  (-0.422,0.005)  p=0.06 | -0.217  (-0.420,-0.014)  p=0.04 |
| Thigh Muscle Area | 0.032  (-0.036,0.100)  p=0.35 | 0.030  (-0.039,0.099)  p=0.38 | 0.025  (-0.045,0.096)  p=0.47 | 0.026  (-0.044,0.096)  p=0.47 | 0.025  (-0.043,0.093)  p=0.46 |
| Added Variable | -2.75  (-7.11,1.61)  p=0.21 | -1.26  (-5.54,3.02)  p=0.56 | 0.421  (-3.91,4.75)  p=0.85 | -0.966  (-6.09,4.16)  p=0.71 | 0.013  (-0.025,0.051)  p=0.50 |
|  | 3MS |  |  |  |  |
|  | Model1+Race | Model1+HTN | Model 1+DM | Model1+APOe4 | Model1+PA |
| Thigh Myosteatosis | -0.145  (-0.253,-0.037)  p=0.01 | -0.134  (-0.240,-0.028)  p=0.01 | -0.143  (-0.251,-0.035)  p=0.01 | -0.142  (-0.250,-0.034)  p=0.01 | -0.140  (-0.245,-0.036)  p=0.01 |
| Thigh Muscle Area | 0.039  (0.004,0.076)  p=0.03 | 0.041  (0.005,0.076)  p=0.02 | 0.039  (0.005,0.073)  p=0.03 | 0.039  (0.006,0.071)  p=0.02 | 0.034  (-0.001,0.069)  p=0.06 |
| Added Variable | -0.592  (-3.25,2.06)  p=0.66 | -1.87  (-4.19,0.460)  p=0.11 | 0.466  (-1.82,2.75)  p=0.68 | 0.067  (-2.47,2.60)  p=0.96 | 0.020  (-0.001,0.040)  p=0.06 |

HTN= hypertension, DM= diabetes mellites, APOe4= presence of an APOe4 allele, PA= total physical activity

Supplementary Table 2: Sensitivity analyses evaluating the impact of other measures of adiposity on the association between thigh myosteatosis and cognitive performance, as measured by the Digit Symbol Substitution Test (DSST) and Modified Mini-Mental Status Exam (3MS). The table presents the correlation coefficient, 95% confidence interval, and p-value for thigh myosteatosis and each added measure of adiposity in relation to cognitive outcomes.

| Variable | Models | | |
| --- | --- | --- | --- |
|  | DSST |  |  |
|  | Model1+BMI | Model 1+VF | Model1+SF |
| Thigh Intermuscular Fat Area | -0.251  (-0.496,-0.005)  p=0.04 | -0.217  (-0.423,-0.007)  p=0.04 | -0.219  (-0.041,0.095)  p=0.06 |
| Thigh Muscle Area | 0.017  (-0.060,0.094)  p=0.66 | 0.020  (-0.051,0.090)  p=0.58 | 0.027  (-0.041,0.095)  p=0.43 |
| Added Variable | 0.312  (-0.393,1.02)  p=0.38 | 0.001  (-0.028,0.031)  p=0.91 | 0.002  (-0.041,0.046)  p=0.92 |
|  |  |  |  |
|  | 3MS |  |  |
|  | Model1+BMI | Model 1+VF | Model1+SF |
| Thigh Muscle Adiposity | -0.151  (-0.286,-0.017)  p=0.03 | -0.142  (-0.254,-0.031)  p=0.01 | -0.151  (-0.268,-0.034)  p=0.01 |
| Thigh Muscle Area | 0.038  (-0.004,0.079)  p=0.08 | 0.038  (0.001,0.076)  p=0.04 | 0.039  (0.003,0.075)  p=0.04 |
| Added Variable | 0.277  (-0.119,0.673)  p=0.17 | -0.001  (-0.016,0.015)  p=0.90 | 0.003  (-0.021,0.027)  p=0.80 |

BMI= Body Mass Index (kg/m2), VF= abdominal visceral fat area, SF= thigh subcutaneous fat area
